# Transcriptome Immune Analysis of the Invasive Beetle *Octodonta nipae* (Maulik) (Coleoptera: Chrysomelidae) Parasitized by *Tetrastichus brontispae* Ferrière (Hymenoptera: Eulophidae)

**DOI:** 10.1371/journal.pone.0091482

**Published:** 2014-03-10

**Authors:** Baozhen Tang, Jun Chen, Youming Hou, E. Meng

**Affiliations:** 1 Fujian Provincial Key Laboratory of Insect Ecology, Department of Plant Protection, Fujian Agriculture and Forestry University, Fuzhou, Fujian, P. R. China; 2 Key Laboratory of Integrated Pest Management on Crops in Fujian-Taiwan, Ministry of Agriculture, Fuzhou, Fujian, P. R. China; Uppsala University, Sweden

## Abstract

The beetle *Octodonta nipae* (Maulik) (Coleoptera: Chrysomelidae) is a serious invasive insect pest of palm plants in southern China, and the endoparasitoid *Tetrastichus brontispae* Ferrière (Hymenoptera: Eulophidae) is a natural enemy of this pest that exhibits great ability in the biocontrol of *O. nipae*. For successful parasitism, endoparasitoids often introduce or secrete various virulence factors to suppress host immunity. To investigate the effects of parasitization by *T. brontispae* on the *O. nipae* immune system, the transcriptome of *O. nipae* pupae was analyzed with a focus on immune-related genes through Illumina sequencing. *De novo* assembly generated 49,919 unigenes with a mean length of 598 bp. Of these genes, 27,490 unigenes (55.1% of all unigenes) exhibited clear homology to known genes in the NCBI nr database. Parasitization had significant effects on the transcriptome profile of *O. nipae* pupae, and most of these differentially expressed genes were down-regulated. Importantly, the expression profiles of immune-related genes were significantly regulated after parasitization. Taken together, these transcriptome sequencing efforts shed valuable light on the host (*O. nipae*) manipulation mechanisms induced by *T. brontispae*, which will pave the way for the development of novel immune defense-based management strategies of *O. nipae*, and provide a springboard for further molecular analyses, particularly of *O. nipae* invasion.

## Introduction

The nipa palm hispid beetle, *Octodonta nipae* (Maulik) (Coleoptera: Chrysomelidae), which is native to Malaysia, is currently wreaking havoc in southern China [1]. The beetles attack young leaf fronds of various palm plants [2], causing significant palm losses to the ornamental palm industry in China each year. The behaviors of *O. nipae*, such as feeding and dwelling in the tightly furled fronds and trunk fibers [2], together with the high stems of palm plants make traditional chemical control ineffective. These results emphasize the necessity for the development of innovative, alternative, and effective management strategies. Although *Metarhizium* can severely infect *O. nipae* [3], the application of *Metarhizium* to manage this beetle is still under investigation. Inspiringly, *Tetrastichus brontispae* Ferriere (Hymenoptera: Eulophidae), a gregarious and koinobiont endoparasitoid, exhibits an enhanced ability in the biocontrol of *O. nipae* pupae [4]. *T. brontispae* manipulates the physiology and biochemistry of *O. nipae* pupae to create a milieu suitable for its progeny development via a variety of different mechanisms, and deciphering these mechanisms is beneficial to execute effective pest control strategies.

Hymenopteran endoparasitoids deposit their eggs into the host insect haemocoel, whose larvae feed on the host until its death [5]. To effectively parasitize, endoparasitoids during oviposition introduce or secrete various virulent factors, such as polydnaviruses (PDVs), venoms, and virus-like particles (VLPs) into the haemocoel of their host insect [6], [7]. These secretory products circumvent or impair the host immune response, including humoral and cellular immune responses, which are associated with a wide array of immune-related genes. These genes can be classified into four categories: (1) pathogen recognition receptors (PRRs), (2) extracellular signal transduction and modulatory enzymes, such as serine proteinases (SPs), their non-catalytic homologs (SPHs), and serine proteinase inhibitors, (3) receptors mediating intracellular signaling pathways and regulation, and (4) effector response systems, such as antimicrobial peptides, phenoloxidase (PO)-dependent melanization system, and genes associated with apoptosis [8]–[10]. In addition to inducing immunosuppression, these secretory products also alter host development, endocrine physiology (often referred to as ecdysteroids and juvenile hormones), and nutritional physiology [11]–[13].

The advent of next-generation sequencing technologies (NGS) combined with bioinformatics tools can generate extensive data on the alterations in the host’s gene expression upon a parasitization challenge at a global level, which is invaluable particularly in the absence of a sequenced genome. Etebari et al. [14] used an Illumina-based transcriptome technique to investigate immune-related genes combined with developmental- and non-immune metabolism-related genes in *Plutella xylostella* parasitized by *Diadegma semiclausum*. Zhu et al. applied transcriptome and digital gene expression (DGE) analyses through Illumina sequencing to investigate immunity-related genes in the yellow mealworm beetle, *Tenebrio molitor*, parasitized by *Scleroderma guani* [15]. As previously described, the transcriptional responses of a host to a parasitoid have been investigated in some host-parasitoid systems; however, the host manipulation by the parasitoid is species-specific [16], and the molecular mechanisms underlying the *O. nipae*-*T. brontispae* immune system have not yet been explored. In addition, the genetic resources for *O. nipae* are surprisingly scarce, which does not appear to reconcile with its critical invasion. Thus, in this study, we used Illumina/Solexa next-generation sequencing to obtain a global transcriptome of *O. nipae* and a comprehensive view of the immune-related genes that are differentially expressed in non-parasitized versus parasitized *O. nipae* pupae. These transcriptome sequencing efforts shed valuable light on the host (*O. nipae*) manipulation mechanisms by *T. brontispae*, which are advantageous to effectively control *O. nipae*, and provide a springboard for further molecular analyses, specifically on *O. nipae* invasion.

## Materials and Methods

### Insects and Parasitization

*Octodonta nipae* were maintained at 25±1°C, 85±5% RH, and a 12∶12 light: dark (L: D) photoperiod on the central leaves of fortunes windmill palm, *Trachycarpus fortunei* (Hook), as previously described [1]. *Tetrastichus brontispae* were cultured with one-day-old *O. nipae* pupae as hosts (the day of newly exuviated pupae was assigned as one-day-old), and adult parasitoids were fed with a 10% sucrose solution. One-day-old *O. nipae* pupae were exposed to newly mated *T. brontispae* adults until parasitization was observed. The attacked pupae were collected individually in a plastic tube (2 ml) and allowed to develop under the same conditions. RNA samples were obtained from parasitized *O. nipae* pupae at different time intervals post-parasitization, i.e., 6, 12, 24, 36, 48, 72, 96, and 120 h post-parasitization. RNA samples from non-parasitized pupae were collected simultaneously as controls. Twenty pupae were collected at each time point.

### cDNA Library Construction and Illumina Sequencing

Two libraries, namely the non-parasitized and the parasitized libraries, were constructed, and each library was completed using pooled RNA with equal amounts from each of the samples of the eight different time points. In addition, to gain a comprehensive transcriptome of *O. nipae* (for further molecular analyses specifically on *O. nipae* invasion), pooled mRNA from the *O. nipae* egg, larvae, pupae, and adult females and males was prepared, and the library (denoted mixed library) was constructed. Total RNA was isolated using the TRIzol reagent (Invitrogen, Carlsbad CA, USA) according to the manufacturer’s instructions and treated with DNase Ι. RNA sample concentration and integrity were determined using a 2100 Bioanalyzer (Agilent Technologies). Poly-A-containing mRNAs were enriched using oligo (dT) magnetic beads, fragmented with RNA Fragmentation Reagent, and subjected to the procedure: first- and second- strand cDNA synthesis, purification, end reparation, single nucleotide A addition, ligation of adapters, purification of ligated products, and PCR amplification for cDNA template enrichment. The cDNA library was qualified and quantified with an Agilent 2100 Bioanalyzer and ABI StepOnePlus Real-time PCR system, respectively, and then sequenced for 90 bp using the Illumina HiSeq™ 2000 platform at the Beijing Genomics Institute (BGI, Shenzhen, China).

### Transcriptome Analysis

After filtering out the sequencing adapters, unknown nucleotides larger than 5% and low quality reads, the resulting clean reads were assembled using Trinity [17]. The resulting sequences from Trinity were output as unigenes. The clean data sets containing our sequences and their quality scores are available at the NCBI Short Read Archive (SRA) with accession number SRP034648. For annotation, unigenes were aligned by BLASTx with an E-value cut-off of 10^−5^ against the NCBI non-redundant (nr), Swiss-Prot, Kyoto Encyclopedia of Genes and Genome (KEGG, http://www.genome.jp/kegg/), and Cluster of Orthologous Groups (COG, www.ncbi.nlm.nih.gov/COG) protein databases. Gene Ontology (GO) annotation of unigenes was analyzed using the Blast2Go software [18], and GO functional classification for all unigenes was performed using the WEGO software [19]. In addition, unigenes without homology to these databases were forecast for their translation direction and open reading frames (ORF) using the ESTScan software [20]. In the absence of *O. nipae* and *T. brontispae* genome sequences, we discarded the annotations that showed similarity to hymenopteran genes, and tried to utilize the annotations that were the most closely related to coleopteran genes in the parasitized library.

### Differentially Expressed Gene (DEG) Analysis

The relative transcript abundance in the non-parasitized and parasitized *O. nipae* pupae was output as FPKM (Fragments Per Kilobase per Million fragments) values according to Mortazavi et al. [21]. Differentially expressed genes (DEGs) between non-parasitized and parasitized *O. nipae* pupae were identified on the basis of the rigorous algorithm, i.e., false discovery rate (FDR)≤0.001 and absolute value of log_2_Ratio≥1, and then subjected to GO functional and KEGG pathway enrichment analyses. For GO enrichment analysis, the calculated *p*-value from the hypergeometric test underwent Bonferroni Correction, and the GO terms with the corrected *p*-value≤0.05 were significantly enriched in all DEGs. For pathway enrichment analysis, pathways with *Q*-value≤0.05 after the multiple testing correction were significantly enriched in all DEGs.

### Quantitative Real-time PCR (qRT-PCR) Validation

To confirm the RNA-seq results, ten randomly selected genes were subjected to qRT-PCR analysis using three replicates. The RNA samples were collected as described above for the transcriptome profiles. Furthermore, for the temporal expression profiles of some DEGs after parasitization, RNA samples at different time points (6, 12, 24, 36, 48, 72, 96, and 120 h post-parasitization) were collected individually.

Total RNA was extracted as previously described and subjected to the Thermo Scientific Verso cDNA Kit (Thermo Fisher Scientific Inc., Waltham MA, USA), where the RT enhancer can remove contaminating DNA and eliminate the need for DNase I treatment. Next, qRT-PCR was performed in triplicate using the Power SYBR Green Master Mix Kit (Invitrogen) with a 20 µl reaction volume containing 250 nM primer (Table S1) and 100 ng of cDNA in an ABI 7500 System. The *Octodonta nipae* ribosomal protein S3 was used as a reference gene [10]. The standard curve of each gene was prepared by serial dilutions (10×) of the cDNA samples. The qRT-PCR profile was performed at 95°C for 10 min, followed by 40 cycles of 95°C for 15 s and 60°C for 1 min, and finally with a dissociation step. All calculations were performed using the accompanying ABI 7500 system software. Data analysis was performed by one-way ANOVA and Tukey’s test using GraphPad InStat (GraphPad Software Inc., San Diego CA, USA).

## Results and Discussion

### Illumina Sequencing and *de novo* Assembly

**Figure 1:**
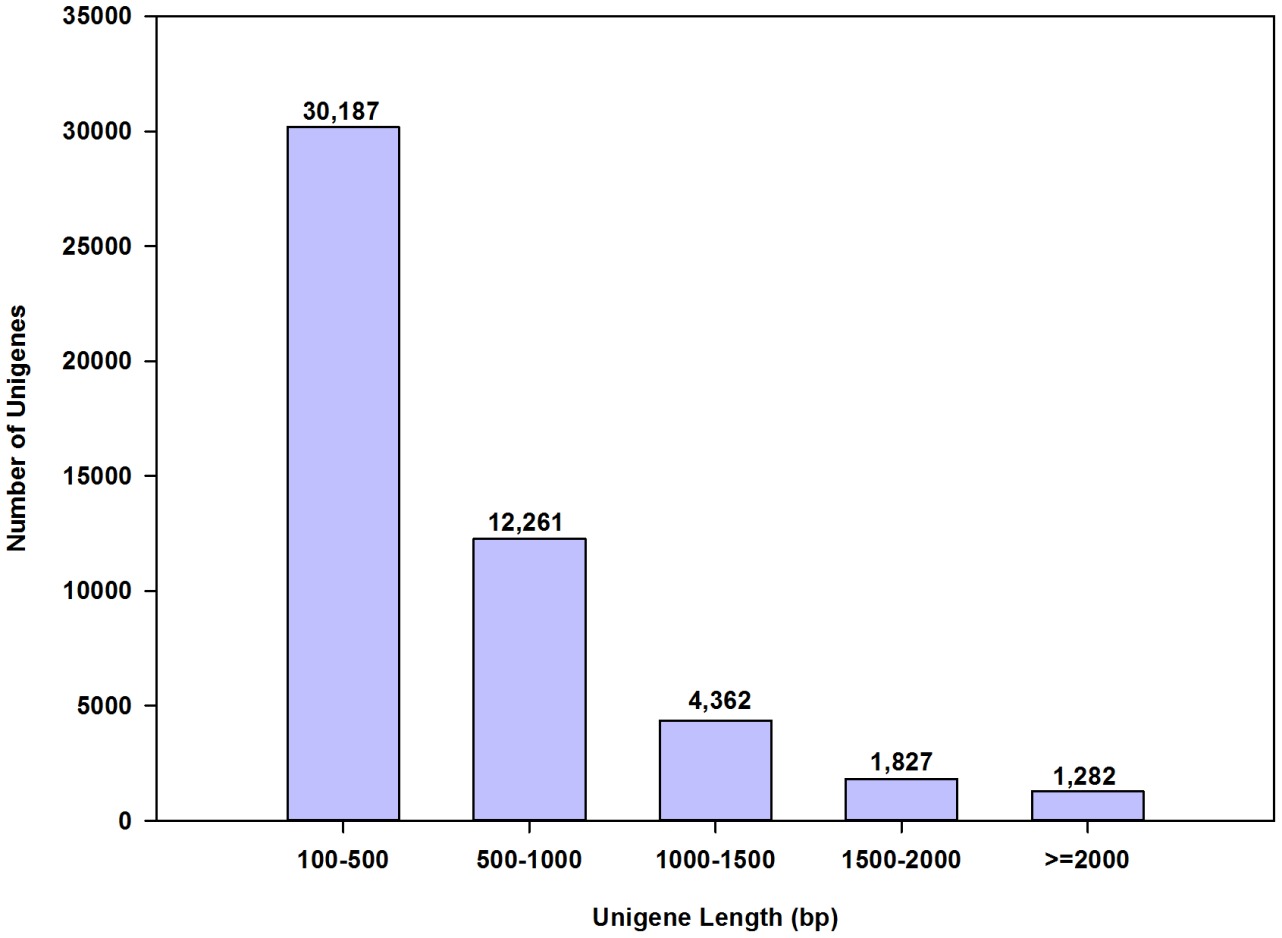
Length distribution of unigenes in assembled *Octodonta nipae* transcriptome. *De novo* assembly produced 49,919 unigenes beteween 100 and 2000 bp in length. The x and y-axes represent the length of unigenes and the number of unigenes in a corresponding length, respectively.

RNA-seq deep sequencing analysis generated approximately 26.5, 34.5, and 33.7 million paired-end reads, which are equivalent to 4, 5, and 5 Gb of data, from the non-parasitized, parasitized, and mixed libraries, respectively. To obtain a comprehensive *O. nipae* transcriptional profile, the total clean reads from the non-parasitized and mixed libraries were combined. *De novo* assembly produced 93,375 contigs with a mean length of 357 bp (Table 1). These contigs were further assembled into 49,919 unigenes with an average size of 598 bp, including 7,471 unigenes (14.96%) over 1000 bp in length (Figure 1). The N50 lengths of the contigs and unigenes were 704 and 795 bp (Table 1), respectively. The mean length of the unigenes in the present assembly results was longer than those from *Tomicus yunnanensis* (355 bp) and *T. molitor* (424 bp) [15], [22], which was most likely due to our increased sequence depth (5 Gb), and can be beneficial for BLAST search and functional annotation.

**Table 1:** Illumina sequencing and assembly summary of the *Octodonta nipae* transcriptome.

Sequencing Parameters	Number
Total reads	67,551,734
Total nucleotides (bp)	6,079,656,060
Q20 percentage (%)*	96.79
N percentage (%)**	0.00
GC percentage (%)	41.99
Number of contigs	93,375
Mean length of contigs (bp)	357
N50 of contig set (bp)***	704
Number of unigenes	49,919
Mean length of unigenes (bp)	598
N50 of unigene set (bp)	795

*Q20 percentage: Percentage of nucleotide error rate under 0.01.

**N: Uncertain base in the output sequencing data.

***N50: Median length of all contigs or unigenes.

### Functional Annotation and Classification

**Figure 2:**
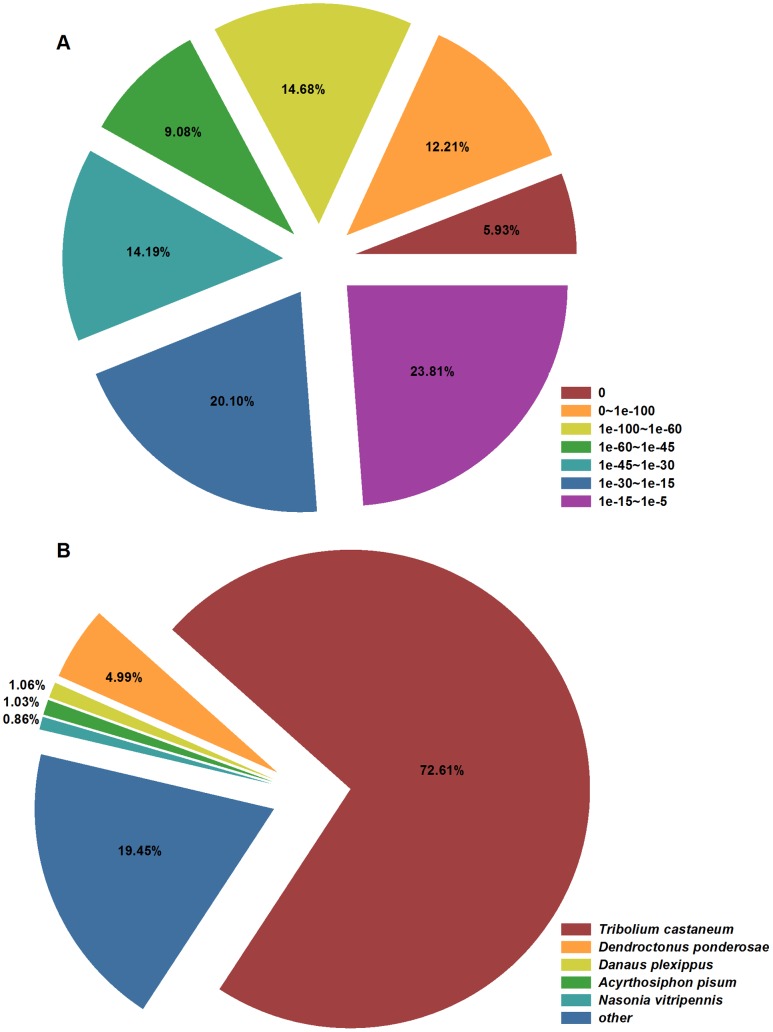
E-value and species distributions of the top BLASTx hits. The BLASTx search was performed against NCBI non-redundant protein database with an E-value cut-off of 10^−5^. A: E-value distribution. B: Species distribution.

For functional annotation, all unigenes were aligned to the GenBank protein databases with a cut-off E-value of 10^−5^ using BLASTx. Using this approach, 27,490 unigenes (55.1% of all unigenes) returned above the cut-off value, indicating that 44.9% (22,429 unigenes) of the total unigenes had no clear homology to known genes. This low annotated percentage was most likely attributed to the deficiency of the *O. nipae* genome (due to the deficiency of the *O. nipae* genome, some transcripts derived from the untranslated regions or non-conserved domains can’t be annotated). The E-value distribution of the top hits in the nr proteins database showed that 11,182 unigenes (41.9%) had significant matches (<1.0E-45), whereas 58.1% of the matched unigenes had E-values that ranged from 1.0E-5 to 1.0E-45 (Figure 2A). For species distribution, most of the unigene sequences (72.6%) matched best to proteins from the red flour beetle (*Tribolium castaneum*), followed by the mountain pine beetle (*Dendroctonus ponderosae*) (5.0%), monarch butterfly (*Danaus plexippus*) (1.1%), pea aphid (*Acyrthosiphon pisum*) (1.0%), and *Nasonia vitripennis* (0.9%; Figure 2B). The present results were consistent with the analyses of other beetle transcriptomes, which showed that 87.9%, 71.6%, and 62.5% of the sequences of *D. ponderosae*, *T. molitor*, and *T. yunnanensis*, respectively, exhibited the highest homology to *T. castaneum* proteins [15], [22], [23]. These high values were expected due to the substantial genome sequences of *T. castaneum* in NCBI.

**Figure 3:**
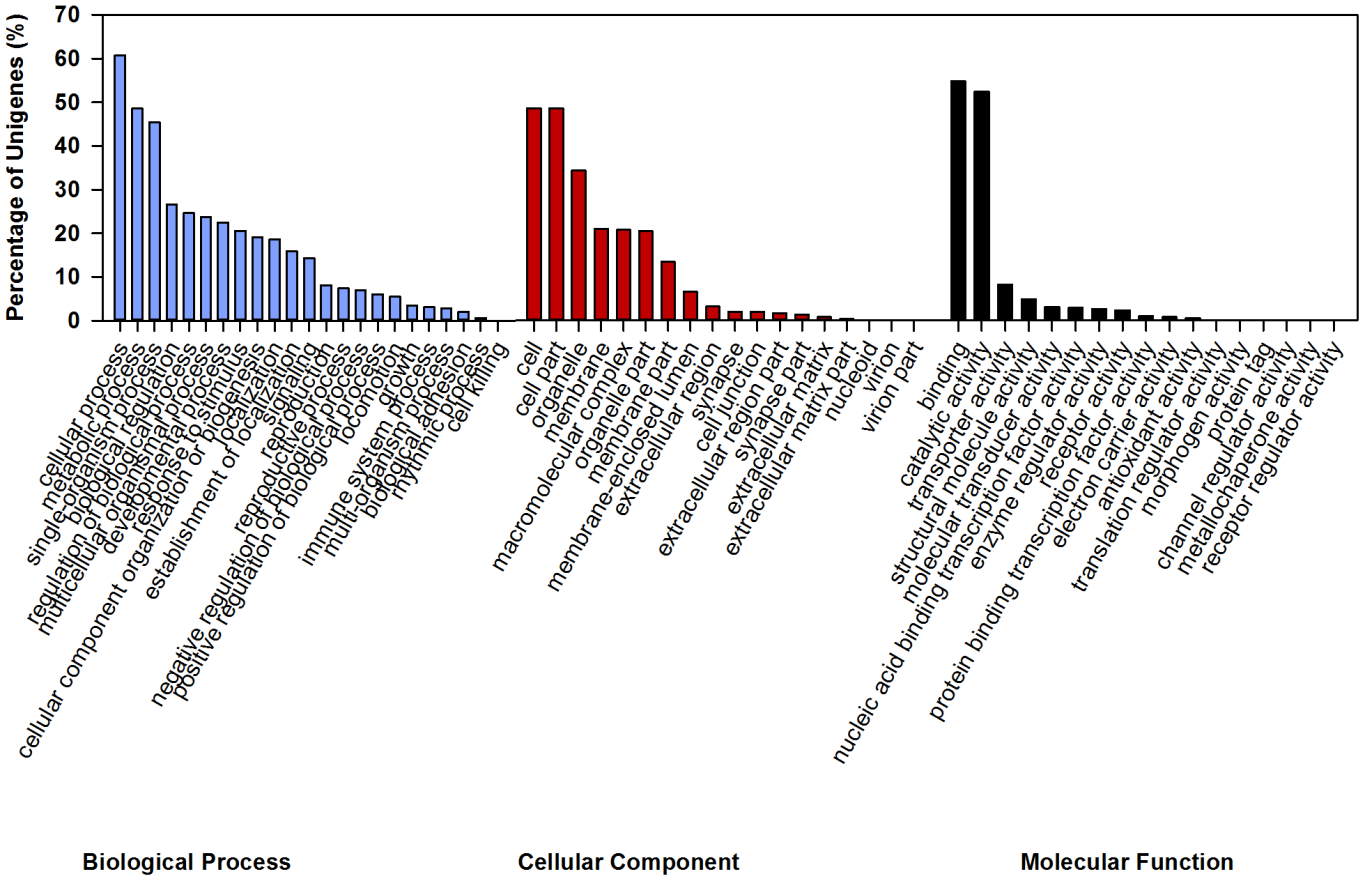
Gene ontology (GO) classification of *Octodonta nipae* unigenes after BLASTx search. Histogram presentation of the GO annotation was generated using WEGO software. A total of 13,031 unigenes were assigned at the second level to three GO ontologies: biological process, cellular component, and molecular function. The y-axis indicates the percentage of a certain GO term within each ontology. One unigene could be assigned to more than one GO term.

**Figure 4:**
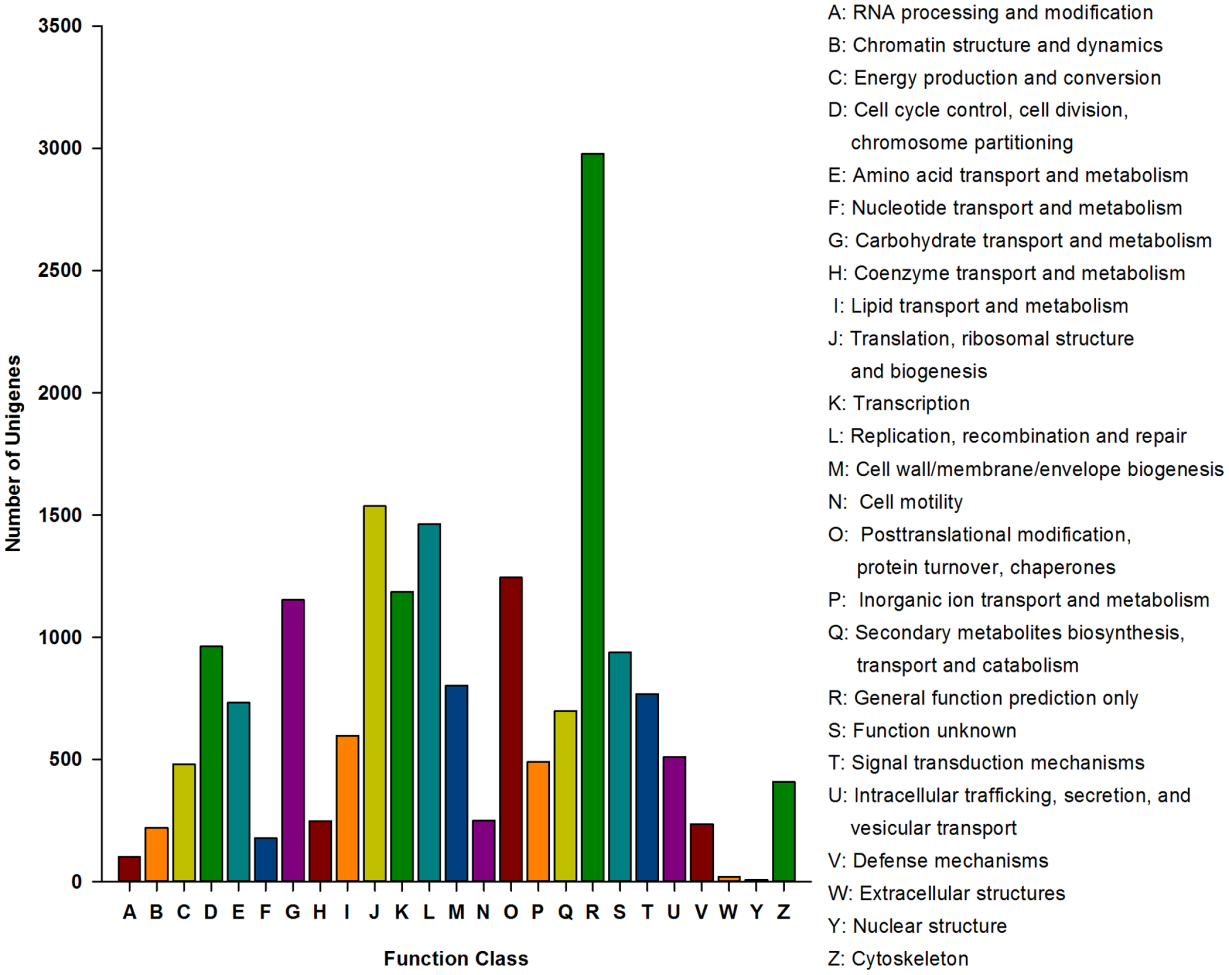
Clusters of orthologous groups (COG) classification of *Octodonta nipae* unigenes after BLASTx search. A total of 8,790 proteins were aligned to the COG protein database and classified functionally into 25 classes. Each function class is denoted by different capital letters under the x-axis. The y-axis represents the number of unigenes in a corresponding function class.

GO analyses were used to identify the potential functions of the predicted proteins. A total of 13,031 unigenes were annotated and assigned to GO terms, which consisted of three main categories: biological process, cellular component and molecular function (Figure 3). Among these GO terms, the most abundant groups were cellular process (7922 unigenes) and metabolic process (6326) for the biological process category, cell (5884) and cell part (5884) for the molecular component category, and binding (6632) and catalytic activity (6348) for the molecular function category. These results indicated the importance of cell communication, metabolic activities, cellular structure, and molecular function in the life cycle of *O. nipae*. Moreover, to further predict the putative protein functions, a COG analysis was performed. Overall, 8,790 unigenes, less than the GO results, were annotated and had a COG classification (Figure 4). Among these 25 COG categories, the cluster of “general function prediction only” was the largest group (2,979, 33.9%), followed by “translation, ribosomal structure, and biogenesis” (1,536, 17.5%) and “replication, recombination, and repair” (1,464, 16.6%). Only 6 and 20 unigenes existed in the “nuclear structure” and “extracellular structures” clusters, respectively, which represented the least groups.

### Enrichment Analysis of DEGs

**Figure 5:**
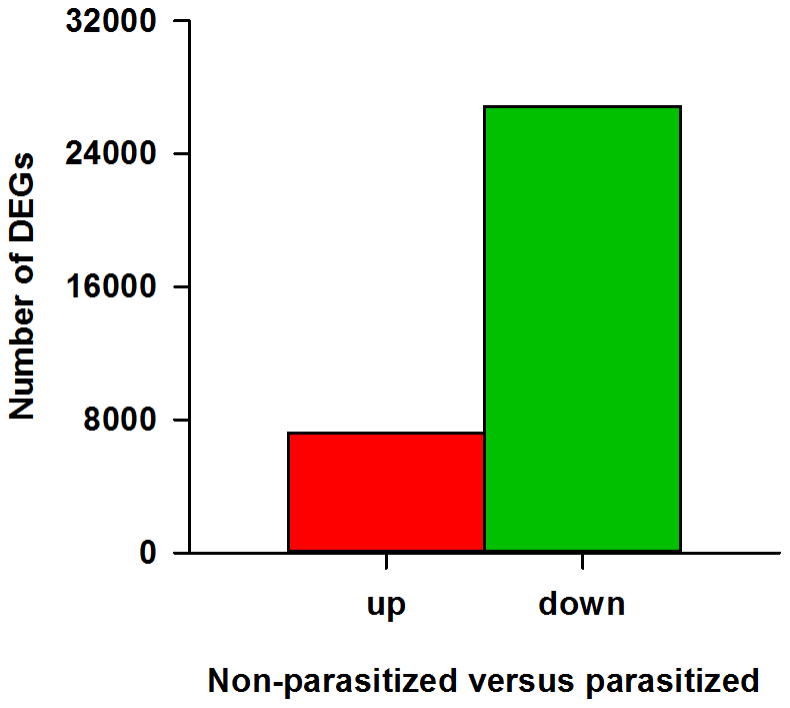
Differential expression analyses between non-parasitized (NP) and parasitized (P) *Octodonta nipae* pupae. The number of up- and down-regulated differentially expressed genes between NP and P libraries was summarized.

Our analyses demonstrated that parasitization by *T. brontispae* exhibited a significant effect on the transcriptome profile of *O. nipae* pupae, and most of these differentially expressed genes (DEGs) were down-regulated (Figure 5). GO analysis revealed that the DEGs were mainly categorized in cellular process and metabolic process with respect to the biological process cluster, in cell and cell part with respect to the cellular component cluster, and in binding and catalytic activity with respect to the molecular function cluster (Figure S1). In total, 59, 42, and 28 GO terms were significantly enriched (*P* value <0.05) in the biological process, cellular component and molecular function categories, respectively (Table S2). For KEGG enrichment analysis, a total of 18,010 unigenes were assigned to 258 KEGG pathways. Among these, 29 pathways were significantly enriched with *Q* value <0.05 (Table S3). Metabolic pathways (2703), RNA transport (699), regulation of actin cytoskeleton (646), and focal adhesion (616) were the major enrichment pathways (Table S3). Taken together, these enrichment analyses indicated that metabolic and cell activities played vital roles in the *O. nipae* response to parasitism.

### Effect of Parasitism on the Transcription of Host Immune-related Genes

When encountering foreign agents, such as bacteria, fungi, virus, and protozoa, insects initiate their innate immune response using pattern-recognition receptors (PRRs) to recognize pathogen-associated molecular patterns (PAMPs). PRRs not only serve as pathogen recognition receptors but also function as opsonins, which facilitate phagocytosis as well as serve as initiators of signaling cascades [8]. After parasitization by *T. brontispae*, we found that the transcriptions of PRRs, such as peptidoglycan recognition proteins (PGRPs), β-1,3-glucan recognition proteins (GRPs), scavenger receptors (SRs), C-type lectins (CTLs), galectins, and Down syndrome cell adhesion molecule (Dscam) were regulated in *O. nipae* pupae (Table 2). Previous studies have also highlighted the pivotal roles of these PRRs in parasitoid-host systems. For example, transcriptome analyses showed that the expression levels of both PGRPs and GRPs changed in *T. molitor* pupae and *P. xylostella* larvae after parasitoid attack [14], [15]. Scavenger receptor transcripts of *P. xylostella* were suppressed by parasitoid factors of *D. semiclausum*, and the SR family plays important roles in the innate immune response in *P. xylostella* [24]. C-type lectin gene expression of *Pieris rapae* decreased after exposure to the venom of *Pteromalus puparum*, and the study concluded that the parasitoid might inhibit activation of the host immune response by suppressing the expression of host C-type lectin [25].

**Table 2:** Immune-related genes differentially transcribed in *Octodonta nipae* pupae following parasitization by *Tetrastichus brontispae*.

Gene style	Gene ID	Gene name	Fold*	*P*-value	FDR**
**Peptidoglycan recognition protein**	CL4266.Contig1	Peptidoglycan-recognition protein SC1a/b(*Drosophila melanogaster*)	1.50	2.89E-30	1.62E-29
	CL4266.Contig2	Peptidoglycan-recognition protein LA(*Drosophila melanogaster*)	1.38	1.19E-39	8.24E-39
	CL4266.Contig5	Peptidoglycan-recognition protein LF(*Drosophila melanogaster*)	1.76	2.75E-19	1.12E-18
	CL1556.Contig1	Peptidoglycan-recognition protein LB(*Drosophila melanogaster*)	−11.98	1.46E-16	5.38E-16
	CL4595.Contig1	Peptidoglycan-recognition protein-SC2 (*Tenebrio molitor*)	2.47	1.45E-278	6.65E-277
	Unigene579	Peptidoglycan recognition protein LF (*Tribolium castaneum*)	1.05	1.62E-11	4.75E-11
	Unigene12367	Peptidoglycan-recognition protein-SC2 (*Tenebrio molitor*)	2.33	0	0
	Unigene36297	Peptidoglycan-recognition protein LF(*Drosophila melanogaster*)	1.56	1.59E-12	4.87E-12
	Unigene37578	Peptidoglycan-recognition protein LC(*Drosophila melanogaster*)	1.23	2.89E-60	2.86E-59
	Unigene44957	Peptidoglycan-recognition protein SC1a/b(*Drosophila melanogaster*)	1.64	4.95E-04	8.19E-04
**β-1,3-glucan-recognition protein**	CL3540.Contig1	Beta-1,3-glucan-binding protein (*Tenebrio molitor*)	−9.87	0	0
	Unigene11397	Beta-1,3-glucan-binding protein (*Tenebrio molitor*)	−15.70	0	0
	Unigene42947	Beta-1,3-glucan-binding protein (*Tenebrio molitor*)	−11.08	0	0
**Gram-negative binding protein**	Unigene10867	GNBP1 (*Tenebrio molitor*)	1.39	6.35E-286	3.01E-284
**C-type lectin**	Unigene43298	C-type lectin (*Tribolium castaneum*)	1.12	1.86E-44	1.41E-43
**Galectin**	CL6400.Contig1	Galectin (*Tribolium castaneum*)	1.03	8.33E-06	1.66E-05
	CL9498.Contig1	Galectin 4-like protein (*Tribolium castaneum*)	1.35	3.78E-169	1.06E-167
**Scavenger receptor**	CL6830.Contig1	Scavenger receptor class B (*Tribolium castaneum*)	−9.31	3.28E-218	1.19E-216
	Unigene11059	Scavenger receptor SR-C-like protein (*Tribolium castaneum*)	1.47	5.52E-210	1.92E-208
	Unigene37336	Scavenger receptor protein (*Tribolium castaneum*)	−12.27	3.84E-28	2.04E-27
**Down syndrome cell adhesion** **molecule**	CL769.Contig8	Down syndrome cell adhesion molecule (*Tribolium castaneum*)	1.01	2.21E-08	5.35E-08
	Unigene1504	Down syndrome cell adhesion molecule (*Tribolium castaneum*)	−13.34	8.31E-57	7.81E-56
	Unigene38836	Down syndrome cell adhesion molecule (*Tribolium castaneum*)	1.34	5.78E-83	7.69E-82
	Unigene38837	Down syndrome cell adhesion molecule (*Tribolium castaneum*)	1.31	1.61E-94	2.43E-93
**Antimicrobial peptide**	CL3241.Contig1	Defensin 1 (*Tribolium castaneum*)	−5.73	3.41E-83	4.54E-82
	CL4664.contig1	Defensin (*Sitophilus zeamais*)	−13.05	4.43E-18	1.74E-17
	CL2637.Contig2	Cecropin precursor (*Acalolepta luxuriosa*)	−12.48	1.12E-10	3.14E-10
	CL7916.Contig1	Cecropin precursor (*Acalolepta luxuriosa*)	−15.58	1.74E-81	2.27E-80
	CL888.Contig1	Attacin-B (*Drosophila melanogaster*)	−8.68	1.45E-140	3.30E-139
	CL888.Contig4	Attacin-C (*Drosophila melanogaste*r)	−11.98	1.59E-13	5.16E-13
	Unigene35100	Acaloleptin (*Acalolepta luxuriosa*)	−8.75	0	0
	Unigene43354	I-type lysozyme (*Sitophilus zeamais*)	1.19	8.03E-33	4.79E-32
	Unigene38231	Lysozyme (*Tribolium castaneum*)	−11.94	3.22E-19	1.30E-18
**Serine protease**	CL1688.Contig2	Serine protease P91 (*Tribolium castaneum*)	1.38	3.27E-18	1.29E-17
	CL7076.Contig2	Serine protease H137 (*Tribolium castaneum*)	1.36	4.09E-10	1.11E-09
	CL7311.Contig1	Serine protease H49 (*Tribolium castaneum*)	−11.56	1.26E-11	3.71E-11
	CL8437.Contig2	Serine protease P12 (*Tribolium castaneum*)	1.94	1.43E-13	4.65E-13
	CL9687.Contig2	Serine protease P56 (*Tribolium castaneum*)	−3.66	2.28E-16	8.30E-16
	Unigene11223	Serine protease P40 (*Tribolium castaneum*)	3.2821	6.92E-10	1.84E-09
	Unigene21374	Serine protease P95 (*Tribolium castaneum*)	−11.97	5.73E-09	1.45E-08
	Unigene28906	Serine protease P136 (*Tribolium castaneum*)	1.54	0	0
	Unigene44394	Serine protease P126 (*Tribolium castaneum*)	−12.00	6.44E-10	1.73E-09
**Serpin**	CL5683.Contig2	Serine protease inhibitor (*Sphenophorus levis*)	−14.50	1.13E-96	1.74E-95
	CL6856.Contig1	Serpin peptidase inhibitor 28 (*Tribolium castaneum*)	1.50	0	0
	CL7775.Contig2	Serine protease inhibitor (*Sphenophorus levis*)	−10.71	0	0
	CL8544.Contig3	Serpin 6 (*Tribolium castaneum*)	1.40	8.34E-195	2.70E-193
	Unigene26323	Serine protease inhibitor (*Tribolium castaneum*)	−13.66	6.92E-104	1.14E-102
	Unigene26335	Serpin B6 (*Tribolium castaneum*)	1.43	0	0
	Unigene40035	Serpin 6 (*Tribolium castaneum*)	−7.43	1.43E-287	6.82E-286
	Unigene40036	Serpin 6 (*Tribolium castaneum*)	−17.28	0	0
**Prophenoloxidase**	CL4352.Contig1	Prophenoloxidase (*Tenebrio molitor*)	−8.34	0	0
	Unigene22758	Pro-phenol oxidase subunit 2 (*Tribolium castaneum*)	−12.29	2.99E-52	2.61E-51
	Unigene24667	Pro-phenol oxidase subunit 2 (*Tribolium castaneum*)	−8.79	0	0
**Integrin**	CL5243.Contig1	Integrin alpha-PS2 precursor (*Tribolium castaneum*)	−9.19	1.36E-200	4.53E-199
	CL1642.Contig1	Integrin beta-PS (*Drosophila melanogaster*)	−15.83	0	0
	CL8257.Contig1	Integrin beta-PS (*Drosophila melanogaster*)	−15.25	7.34E-41	5.21E-40
	Unigene35248	Integrin beta-PS (*Drosophila melanogaster*)	−13.13	5.40E-72	6.32E-71
**Tetraspanin**	CL6151.Contig1	Tetraspanin 97e (*Tribolium castaneum*)	−12.13	6.49E-25	3.15E-24
	Unigene10835	Tetraspanin 2A (*Tribolium castaneum*)	1.24	5.34E-22	2.38E-21
	Unigene13168	Tetraspanin D107 (*Tribolium castaneum*)	1.31	7.04E-61	7.03E-60
	Unigene24667	Tetraspanin (*Tribolium castaneum*)	−8.79	0	0
	Unigene31320	Tetraspanin F139 (*Tribolium castaneum*)	−13.84	1.69E-21	7.43E-21
**Talin**	Unigene33815	Talin-1 (*Gallus gallus*)	1.03	2.31E-210	8.02E-209
	Unigene33812	Talin-1 (*Gallus gallus*)	1.07	2.67E-63	2.77E-62
	Unigene33813	Talin-2 (*Mus musculus*)	1.06	3.38E-26	1.70E-25
**Rac1**	Unigene37971	Rho family, small GTP binding protein Rac1 (*Canis lupus*)	−16.05	4.27E-197	1.39E-195
**CDC42**	CL5452.Contig2	CDC42 small effector protein(*Drosophila melanogaster*)	1.33	1.64E-08	4.02E-08
**Guanine nucleotide exchange factor**	Unigene38538	Guanine nucleotide exchange factor(*Tribolium castaneum*)	−7.58	8.13E-193	2.59E-191
	Unigene3566	Rho guanine nucleotide exchange factor 11(*Tribolium castaneum*)	−13.77	5.45E-158	1.41E-156
**Rho GTPase-activating protein**	CL7328.Contig1	Rho GTPase activating protein (*Tribolium castaneum*)	1.27	5.73E-41	4.07E-40
	CL9874.Contig2	Rho GTPase-activating protein 6 (*Tribolium castaneum*)	1.25	1.41E-09	3.71E-09
**Rac GTPase-activating protein**	CL9874.Contig1	Rho GTPase-activating protein 6 (*Tribolium castaneum*)	1.22	1.43E-07	3.28E-07
	Unigene15957	Rac GTPase-activating protein (*Tribolium castaneum*)	1.31	4.24E-62	4.31E-61
	Unigene15956	Rac GTPase-activating protein (*Tribolium castaneum*)	1.03	8.76E-38	5.80E-37
**Cdc42 GTPase-activating protein**	Unigene33605	Cdc42 GTPase-activating protein (*Tribolium castaneum*)	−9.15	0	0
	Unigene33604	Cdc42 GTPase-activating protein (*Tribolium castaneum*)	−7.49	1.60E-120	3.10E-119
**Toll pathway**	Unigene45778	Spatzle (*Tribolium castaneum*)	−10.37	8.61E-05	1.55E-04
	Unigene33984	MyD88 (*Tribolium castaneum*)	1.23	6.26E-05	1.14E-04
	Unigene35059	Pelle (*Tribolium castaneum*)	1.11	5.13E-36	3.27E-35
**IMD pathway**	Unigene40950	Relish (*Tribolium castaneum*)	−8.67	1.97E-139	4.47E-138
**JAk/STAT**	Unigene28796	Domeless (*Tribolium castaneum*)	1.15	2.30E-165	6.25E-164
	Unigene28797	Domeless (*Tribolium castaneum*)	1.14	1.28E-156	3.28E-155
	Unigene37182	Hopscotch (*Tribolium castaneum*)	−11.83	9.98E-10	2.64E-09
**MAPK –JNK-p38 pathway**	Unigene26492	PDGF- and VEGF-related factor 3 (*Tribolium castaneum*)	1.07	7.83E-88	1.10E-86
	Unigene26493	PDGF- and VEGF-related factor 3 (*Tribolium castaneum*)	−6.43	1.15E-28	6.20E-28
	CL8497.Contig1	Eiger (*Tribolium castaneum*)	−13.56	3.67E-121	7.15E-120
	CL8497.Contig2	Eiger (*Tribolium castaneum*)	−13.80	6.96E-119	1.33E-117

*Fold change was calculated as log_2_ P/NP. P: parasitized. NP: non-parasitized.

**FDR: False discovery rate.

Differentially expressed genes were identified on the basis of FDR≤0.001 and the absolute value of log_2_ P/NP≥1.

**Figure 6:**
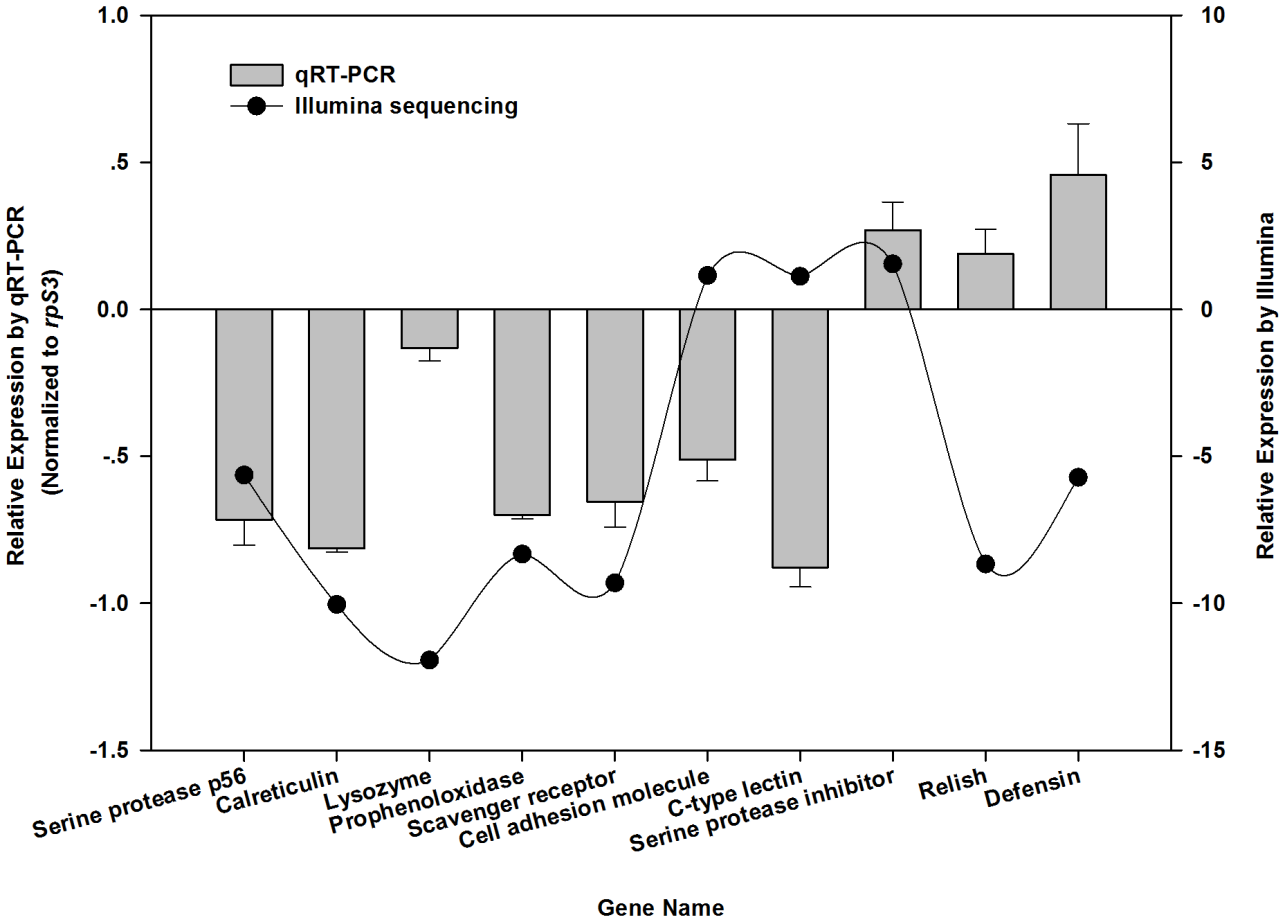
qRT-PCR validation of ten selected genes in *Octodonta nipae* pupae which showed differential expression after parasitization by *Tetrastichus brontispae* on the basis of Illumina sequencing analysis. The relative expression levels of these unigenes were transformed into the log_2_Ratio of parasitized (P) to non-parasitized (NP). The error bars indicate standard deviations of the mean from three independent replications.

AMPs, which are key elements of the innate immunity in insects, also serve crucial roles in opposing pathogenic invasion [26], [27]. In our study, 14 unigenes encoding putative AMPs, such as defensin, cecropin, attacin, acaloleptin, and lysozyme, were down-regulated in *O. nipae* pupae after parasitization compared to the transcription levels in the non-parasitized pupae (Table 2). These results for defensin and lysozyme were also verified by our qRT-PCR analysis (Figure 6). Similarly, cecropin and gloverin in the *Manduca sexta* egg were down-regulated following parasitization by *Trichogramma evanescens* [28]. Barandoc et al. found that parasitization by *Cotesia plutellae* suppressed the expression of cecropin in *P. xylostella* larvae [29]. In contrast, some studies demonstrated that parasitoid challenge induces AMP transcript levels in the host. For example, gloverin, moricin, lysozyme II, and cecropin were up-regulated in *P. xylostella* larvae following *D. semiclausum* attack [14]. Parasitization by *S. guani* enhanced the expression levels of attacin and acaloleptin in *T. molitor* [15]. In addition, it has been reported that leureptin and attacin could not be induced after *T. evanescens* parasitization in the *M. sexta* egg [28]. As previously described, AMPs are diverse in different parasitoid-host systems, and this difference is potentially attributed to the presence of species-specific AMPs together with their marked sequence diversity [10].

Extracellular enzymes involved in melanization, such as serine proteases, serpins, and prophenoloxidase (proPO) (down-regulated), were regulated after parasitization in our study (Table 2). Melanization is thought to play crucial roles in wound healing, encapsulation, sequestration of microorganisms, and production of toxic intermediates [30]–[32]. Etebari et al. reported that transcripts of a serine protease and serpins were up-regulated in *P. xylostella* larvae after parasitization by *D. semiclausum* [14]. In contrast, in the same host parasitized by *C. plutellae*, the protein profile of pxSerpin 2 was suppressed during the course of parasitism [33]. In *M. sexta*, the bracovirus protein Egf1.0 produced by the wasp *Microplitis demolitor* inhibited the PO cascade [34], [35]. Similarly, a serpin LbSPNy highly expressed in the venom of *Leptopilina boulardi* targeted the *Drosophila* PO cascade [36]. The PO cascade is known to be tightly regulated by serine protease and serpins [37], and the regulations of serine protease and serpins are suspected to contribute to an endoparasitoid immune suppressive strategy.

Encapsulation is a major immune response against endoparasitoid eggs that are too large to be phagocytized by individual hemocytes [7], [38]. During this process, hemocyte adhesion and shape change are essential parts of the cellular immune response against parasitoid wasp eggs. In this study, we mainly described two types of central proteins involved in these processes.

Integrins are heterodimeric transmembrane glycoproteins consisting of two non-covalently associated α and β subunits [39]. These proteins are cellular adhesive proteins, and have been elucidated to be involved in hemocyte spreading and encapsulation in insects [40]. For example, in *M. sexta*, the dsRNA targeting three α integrin subunits abolished the encapsulation response to foreign surfaces [41], and the RNAi of integrin β1 significantly suppressed the encapsulation of DEAE-Sephadex beads in larval hemocytes [39]. The expression of integrin α2 and β1 increased when hemocytes bound to a foreign surface or formed a capsule in *Pseudoplusia includens* [42]. The integrin β1 subunit of *Ostrinia furnacalis* was confirmed to play an important role in regulating the spreading of plasmatocytes [40], [43]. In the current study, the transcripts of both α and β subunits were down-regulated in parasitized pupae of *O. nipae* (Table 2). It is likely that *T. brontispae* may suppress the integrin expression levels to interfere with hemocyte spreading and encapsulation. Moreover, the transcript of tetraspanin, an integrin ligand, was also regulated (Table 2). Similarly, tetraspanin D76 was discovered to be associated with the adhesion of hemocytes in *M. sexta* [44]. In addition, the transcriptional levels of integrin signaling molecules, such as talin in *O. nipae* pupae, were also altered (up-regulated) after parasitization by *T. brontispae* (Table 2). Talin is required for integrin function and acts to connect ECM (extracellular matrix)-bound integrins to the actin cytoskeleton in *Drosophila* [45].

Rho GTPases, including Rho, Rac and Cdc42, belong to one family of proteins that are pivotal to many cellular processes, such as cytoskeletal organization, regulation of cellular adhesion, cellular polarity, and transcriptional activation [46], [47]. In *Drosophila melanogaster*, Rac2 was found to be necessary for plasmatocyte spreading and the formation of septate junctions during capsule formation around the parasitoid egg of *L. boulardi* [48]. Furthermore, Rac1 regulated the formation of actin- and focal adhesion kinase (FAK)- rich placodes in hemocytes and was required for the proper encapsulation of *L. boulardi* eggs [49]. Rho GTPases act by cycling between active/GTP-bound and inactive/GDP-bound states [50]. This cycle is regulated by guanine nucleotide exchange factors (GEFs), GTPase-activating proteins (GAPs), and guanine nucleotide dissociation inhibitors (GDIs). GEFs enhance the exchange of GDP for GTP to enable GTPases, GAPs bind to GTPases and the consequent stimulation of GTP hydrolysis negatively regulates the switch. GDIs sequester and solubilize the GDP-bound form to block the GTPase cycle [51]–[53]. Our analysis showed that Rac1 and GEFs transcripts were down-regulated, and the transcripts of Rho-GAPs and Rac-GAPs were up-regulated in the parasitized pupae of *O. nipae* (Table 2). In contrast, Cdc42 was up-regulated and Cdc42-GAPs were down-regulated (Table 2). Due to the diverse roles of Rho GTPases, it is not surprising that the transcripts of the Rho GTPases family and their effectors (regulators) were altered in *O. nipae* pupae after parasitization by *T. brontispae*. However, the mechanisms underlying the distinct changes between Rac (Rho) and Cdc42 should be further investigated.

In addition to the genes that have been described above, other genes related to signal transduction pathways, such as Toll, IMD, JAK-STAT, and JNK-p38, were regulated following parasitization (Table 2). Similarly, in the *Drosophila* larvae, components of the Toll and JAK/STAT pathways were up-regulated after *L. boulardi* attack [54]. In *P. xylostella* larvae parasitized by *D. semiclausum*, the transcription levels of proteins similar to the Toll receptor were up-regulated [14]. In *T. molitor* pupae parasitized by *S. guani*, transcripts associated with the Toll and IMD pathways were affected [15]. Intracellular signaling pathways control the production of effector molecules, and each pathway targets different functional groups [9], [55], [56]. Thus, the regulation of intracellular signal cascades is likely one of the parasitoid wasp infection strategies.

### Quantitative RT-PCR Validation of Transcriptome Analysis

To validate the Illumina expression profiles, ten genes were randomly selected (Figure 6) for qRT-PCR analysis, and the same RNA samples as that for transcriptome profiles were applied. The qRT-PCR results showed that the trends of six out of ten selected genes were similar to those from Illumina sequencing in the up- or down-regulation of the host (Figure 6), whereas the trends of the remaining four genes were inconsistent with the Illumina sequencing data (Figure 6). Given that it was difficult to completely exclude the transcriptome of the endoparasitoid *T. brontispae* from that of the host, the deviation was most likely due to the mixed reads of *O. nipae* and *T. brontispae* obtained from the DEG analysis based on the FPKM method. However, the goal of the present study was to obtain an overview of what occurs after a parasitoid attack, and the deviation may have only a slight effect on our analysis.

**Figure 7:**
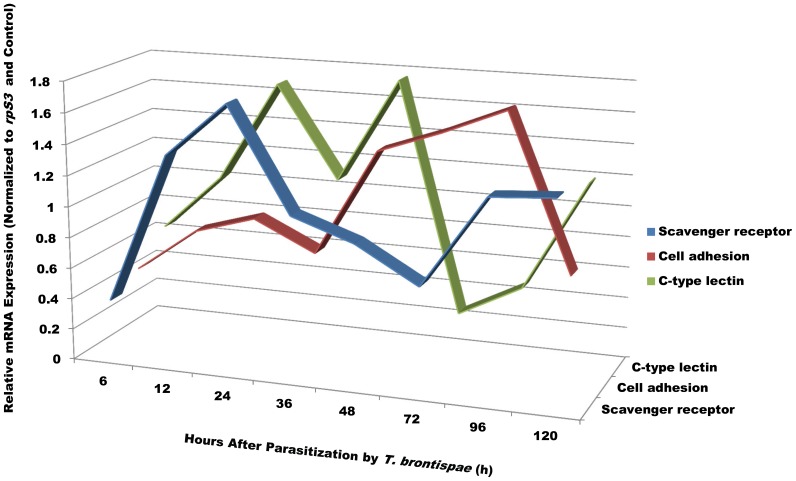
qRT-PCR analysis of expression profiles of three randomly selected genes (scavenger receptor, C-type lectin, and cell adhesion molecule) in *Octodonta nipae* pupae at different time points after parasitization by *Tetrastichus brontispae*. The expression levels were normalized to the *ribosomal protein S3* (reference gene) and the non-parasitized pupae.

Furthermore, to gain insights into the temporal expression profiles of immune-related genes after parasitization, three randomly selected genes were analyzed by qRT-PCR (Figure 7). As expected, the expression levels of the selected genes, scavenger receptor, C-type lectin, and cell adhesion molecule, varied at different periods after parasitization (Figure 7). For example, the expression levels of all three genes were suppressed six hours post-parasitization, increased prior to 24 hours post-parasitization, declined 36 hours post-parasitization, and exhibited distinct patterns in the following hours (Figure 7).

## Conclusions

Overall, our study presents the first global transcriptome of *O. nipae* and, more importantly, an overview of the immune effect of an endoparasitoid wasp on *O. nipae* pupae. The transcriptome profiling data obtained in this study provide a foundation for future molecular analyses, specifically on *O. nipae* invasion. The identified immune-related genes provide an invaluable resource for elucidating the mechanisms underlying the *O. nipae*-*T. brontispae* immune system. Moreover, it will pave the way for the development of novel immune defense-based management strategies of *O. nipae*.

## Supporting Information

Figure S1 **Gene ontology (GO) classification of differentially expressed genes (DEGs) between non-parasitized and parasitized** ***Octodonta nipae*** **pupae.** DEGs between non-parasitized and parasitized *O. nipae* pupae were identified on the basis of false discovery rate (FDR)≤0.001 and absolute value of log_2_Ratio≥1. Histogram presentation of the GO annotation was generated using WEGO software. Genes were assigned at the second level to three GO ontologies: biological process, cellular component, and molecular function. The y-axis indicates the percentage of a certain GO term within each ontology. One unigene could be assigned to more than one GO term.
(TIF)

Table S1 **Primers used for the qRT-PCR analysis.**
(DOCX)

Table S2 **Significantly Gene Ontology (GO) enrichment analysis of differentially expressed genes between non-parasitized and parasitized** ***Octodonta nipae*** **pupae.** GO terms with the corrected *P* value <0.05 were significantly enriched.
(XLSX)

Table S3 **Kyoto Encyclopedia of Genes and Genome (KEGG) enrichment analysis of differentially expressed genes between non-parasitized and parasitized** ***Octodonta nipae*** **pupae.** Pathways with *Q*-value<0.05 were significantly enriched.
(XLSX)
