# Supplementary figures and images for: Transcriptome Immune Analysis of the Invasive Beetle Octodonta nipae (Maulik) (Coleoptera: Chrysomelidae) Parasitized by Tetrastichus brontispae Ferrière (Hymenoptera: Eulophidae)
Source: PLoS One. 2014 Mar 10;9(3):e91482. doi: 10.1371/journal.pone.0091482 (PMC3948882; doi:10.1371/journal.pone.0091482)

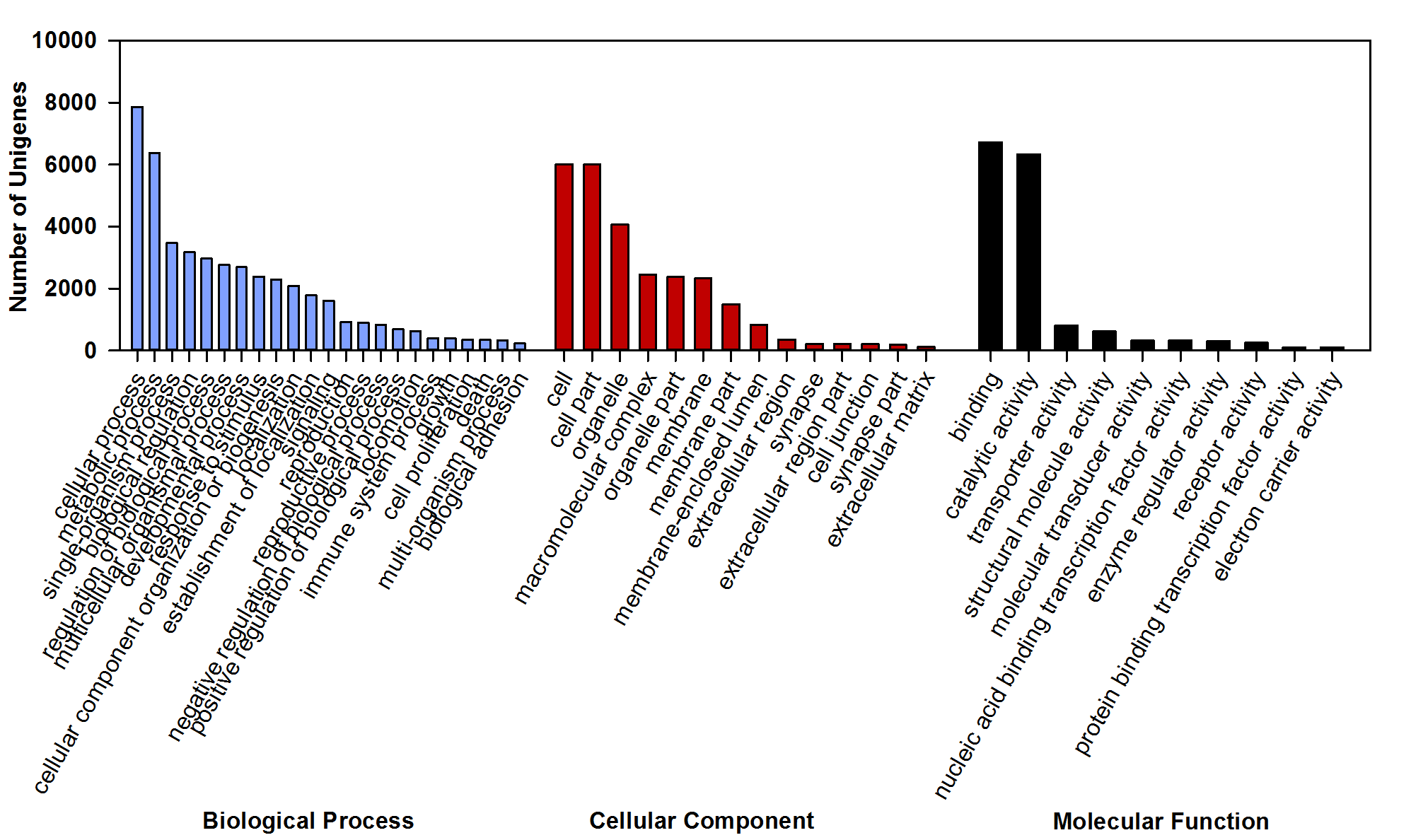

Supplement: Figure S1 — Gene ontology (GO) classification of differentially expressed genes (DEGs) between non-parasitized and parasitized Octodonta nipae pupae. DEGs between non-parasitized and parasitized O. nipae pupae were identified on the basis of false discovery rate (FDR)≤0.001 and absolute value of log2Ratio≥1. Histogram presentation of the GO annotation was generated using WEGO software. Genes were assigned at the second level to three GO ontologies: biological process, cellular component, and molecular function. The y-axis indicates the percentage of a certain GO term within each ontology. One unigene could be assigned to more than one GO term. (TIF) [file pone.0091482.s001.tif]
