# Supplementary material for: Transcriptome Immune Analysis of the Invasive Beetle Octodonta nipae (Maulik) (Coleoptera: Chrysomelidae) Parasitized by Tetrastichus brontispae Ferrière (Hymenoptera: Eulophidae)
Source: PLoS One. 2014 Mar 10;9(3):e91482. doi: 10.1371/journal.pone.0091482 (PMC3948882; doi:10.1371/journal.pone.0091482)
Supplement: Table S1 — Primers used for the qRT-PCR analysis. (DOCX) [file pone.0091482.s002.docx]

**Table S1.** Primers used for the qRT-PCR analysis.

| Gene Name | Forward Primer (5’-3’) | Reverse Primer (5’-3’) |
| --- | --- | --- |
| Ribosomal protein S3 | GACGGTGTCTTCAAAGCTGA | ATTTCTGTACGTGTCGGGGT |
| Serine protease P56 | CGGTTGGTGGAAAGTGTCAG | CCCTCGTTGTCCAGCTTCTA |
| Serine protease inhibitor 28 | TCGCCTTAGTGATAGCGTGT | ACAGGGCTAGGGAAAACTCC |
| C-type lectin | TGGACTAAACCCCGGTCTTC | AGTTTCAAACACCTGCCGTC |
| Scavenger receptor | GCCCAGAGTATTCCTTCCGA | TTGGCGTACTCTGGAAGGTT |
| Relish | GGTTCAACTGCTAGGCTTCG | GGTGGCATACTTCAGCACAG |
| Defensin | TGTGACGTTCTCAGTGCAGA | CAGTAACCACCAGACTTCCCT |
| Lysozyme | ACGAATTTCCACCTTTGCCA | AACTCGTGTCACCAATGCTG |
| Calreticulin | ATGCTCGCTTTTATGCCCTC | TGACATATCCACCTCCGCAA |
| Cell adhesion molecule | ATGGGATATGGAGGCAGACG | ACCTACTGCAGCACACTCAA |
| Prophenoloxidase | TATTGGTGTGCGCAATGTGG | GGCAACAGAAAGAGCGTAGT |
